# In vitro detection of in vitro secondary mechanisms of genotoxicity induced by engineered nanomaterials

**DOI:** 10.1186/s12989-019-0291-7

**Published:** 2019-02-13

**Authors:** Stephen J. Evans, Martin J. D. Clift, Neenu Singh, John W. Wills, Nicole Hondow, Thomas S. Wilkinson, Michael J. Burgum, Andy P. Brown, Gareth J. Jenkins, Shareen H. Doak

**Affiliations:** 1 In Vitro Toxicology Group, Institute of Life Science, Swansea Univeristy Medical School, Swansea University, Singleton Park, Swansea, SA2 8PP Wales, UK; 2 Faculty of Health Sciences and Life Sciences, School of Allied Health Sciences, De Montfort University, The Gateway, Leicester, LE1 9BH UK; 3 Department of Veterinary Medicine, School of Biological Sciences, University of Cambridge, Madingley Road, Cambridge, CB3 0ES UK; 4 School of Chemical and Process Engineering, University of Leeds, Leeds, LS2 9JT UK

**Keywords:** Nanoparticles, Nano(geno)toxicology, Secondary genotoxicity, Immune cells, In vitro models, Conditioned media, Co-culture models

## Abstract

**Background:**

It is well established that toxicological evaluation of engineered nanomaterials (NMs) is vital to ensure the health and safety of those exposed to them. Further, there is a distinct need for the development of advanced physiologically relevant in vitro techniques for NM hazard prediction due to the limited predictive power of current in vitro models and the unsustainability of conducting nano-safety evaluations in vivo. Thus, the purpose of this study was to develop alternative in vitro approaches to assess the potential of NMs to induce genotoxicity by secondary mechanisms.

**Results:**

This was first undertaken by a conditioned media-based technique, whereby cell culture media was transferred from differentiated THP-1 (dTHP-1) macrophages treated with γ-Fe_2_O_3_ or Fe_3_O_4_ superparamagnetic iron oxide nanoparticles (SPIONs) to the bronchial cell line 16HBE14o^−^. Secondly construction and SPION treatment of a co-culture model comprising of 16HBE14o^−^ cells and dTHP-1 macrophages. For both of these approaches no cytotoxicity was detected and chromosomal damage was evaluated by the in vitro micronucleus assay. Genotoxicity assessment was also performed using 16HBE14o^−^ monocultures, which demonstrated only γ-Fe_2_O_3_ nanoparticles to be capable of inducing chromosomal damage. In contrast, immune cell conditioned media and dual cell co-culture SPION treatments showed both SPION types to be genotoxic to 16HBE14o^−^ cells due to secondary genotoxicity promoted by SPION-immune cell interaction.

**Conclusions:**

The findings of the present study demonstrate that the approach of using single in vitro cell test systems precludes the ability to consider secondary genotoxic mechanisms. Consequently, the use of multi-cell type models is preferable as they better mimic the in vivo environment and thus offer the potential to enhance understanding and detection of a wider breadth of potential damage induced by NMs.

**Electronic supplementary material:**

The online version of this article (10.1186/s12989-019-0291-7) contains supplementary material, which is available to authorized users.

## Introduction

The unique physico-chemical properties of nanomaterials (NMs) have enabled novel applications (either current or in development) in diverse sectors including medicine, cosmetics, agriculture, electronics and aerospace [31]. It has been consistently reiterated that the toxicological profile of a NM is not however comparable to its bulk counterpart due to such unique physico-chemical properties as the ultra-small size and high surface area promote different, and potentially adverse biological interactions. NM size is key in this regard as it may expedite uptake, penetration into tissue and translocation throughout the body [22].

Numerous studies have highlighted the potential of NMs to promote an inflammatory response, cytotoxicity and genotoxicity [14, 28, 38]. All of these toxicological endpoints are of concern, in particular the risk of the induction of DNA damage which could ultimately result in carcinogenesis [24, 42]. The mechanisms for the induction of DNA damage in a single cell type by NMs are categorised as primary direct genotoxicity where a NM interacts directly with the DNA molecule or DNA associated proteins; and primary indirect genotoxicity where the NM does not physically interact with the DNA molecule, but instead damage is induced by the exogenous agent interfering with the action of proteins involved in DNA replication, cell division, DNA fidelity, or via the induction of processes such as oxidative stress or lipid peroxidation. Where a NM is able to affect more than one cell type (e.g. components of the innate immune system and an epithelial cell type), secondary mechanisms of DNA damage maybe induced as a result of NM interaction with one cell type causing downstream secondary genotoxicity in another [16]. Secondary genotoxicity is typically only evident in vivo. The mechanism of damage induced will ultimately be determined both by the NM’s physico-chemical characteristics and the biological environment of the exposure. The lung for instance is one of the key portals of NM entry into the body comprised of multiple cell types including immune cells [11]. If a NM is able to induce a chronic immune response there is a risk of the induction of genotoxicity by secondary mechanisms. This may be initiated by the persistent presence of a foreign material, resulting in the tissue becoming flooded with reactive oxygen species (ROS) and reactive nitrogen species (RNS), causing cellular stress [26].

The principle of secondary genotoxicity promotion by NMs in the lung is supported by a number of in vivo studies. For example, a recent study instilled rutile TiO_2_ particles with either a positive or negative surface charge into the lungs of C57Bl/6J mice (at single exposure doses of 18, 54, 162 μg/mouse; [47]. They observed DNA damage was unaffected by the surface charge of the particles and attributed the response to inflammatory responses caused by cell-cell interactions resulting in secondary genotoxicity.

In comparison, traditional in vitro nano(geno)toxicology studies are typically conducted in unicellular monocultures, which only allow for the detection of primary genotoxicity and are unable to replicate secondary genotoxic mechanisms thought to be responsible for NM-induced DNA damage in vivo [9, 14]. Although in vitro DNA damage assessments of NMs has primarily been focused in single cell mono-cultures various alternative methods have been developed in the assessment of certain toxicological endpoints that permit or replicate the interaction of different cell types. Arguably the most straightforward methodology that can be applied to achieve this is the conditioned medium approach. This technique has typically been applied to investigate the impact of an initial immune response of one particular cell type on a second type [16]. A study by Barlow and colleagues for example, took this approach where the cell culture medium from type II alveolar cells initially treated with carbon black NPs was applied to alveolar macrophages, promoting macrophage chemotaxis [5]. A significant caveat in this approach however, is the lack of *direct* cell-to-cell interactions that occur in vivo.

Such direct cell-to-cell interactions however, can be modelled using an in vitro co-culture system. Co-culture models are typically constructed of two or more cell types including epithelial and immune cells. The application of such test systems to DNA damage assessment are currently highly limited, although various co-culture models have been developed that mimic lung tissue for cytotoxicity, inflammatory and NM uptake assessment [3, 10, 20]. Further development of techniques such as conditioned media treatments and co-culture models will aid in the work to ‘bridge the gap’ between in vivo and in vitro NM genotoxicity assessment [48].

This study aimed to utilise these approaches for the assessment of secondary genotoxic mechanisms in vitro. For this investigation, dextran coated γ-Fe_2_O_3_ and Fe_3_O_4_ ultrafine superparamagnetic iron oxide nanoparticles (dSPIONs) were selected as model NPs. SPIONs may pose a significant risk, via inhalation, in an occupational exposure scenario and have potential for usage in pulmonary drug delivery systems [18]. Furthermore a number of studies have demonstrated the ability of SPIONs to promote genotoxicity both in vivo and in vitro [1, 2, 46]. Furthermore, a study using identical dSPION has previously identified only γ-Fe_2_O_3_ NPs to be genotoxic in mono-cultured human lymphoblast cells [41]. The current study was undertaken by assessing the (pro-)inflammatory and primary indirect genotoxic potential of γ-Fe_2_O_3_ and Fe_3_O_4_ dSPIONs. This was followed by secondary genotoxicity assessment by the in vitro micronucleus assay, in the first instance following exposure of 16HBE14o^−^ to dSPION suspended in an immune cell (dTHP-1 macrophage) conditioned cell culture medium. Finally, a dual cell co-culture model of both 16HBE14o^−^ and dTHP-1 macrophages was constructed to allow physiologically relevant cell-to-cell contact and interactions to occur during exposure to dSPIONs. Cellular uptake of SPIONs without nuclear penetration was demonstrated by electron microscopy of the cells and co-culture sections. By undertaking this investigation, it was hypothesised that by utilising conditioned media treatments and co-culture models’ mechanisms of secondary genotoxicity may be induced, which would be unachievable when using mono-culture systems.

## Results and discussion

This study aimed to develop in vitro models able to evaluate secondary genotoxicity induced by NMs. dSPIONS were used here as test vehicles and the physicochemical characteristics of these particle types are presented in Table 1; and only differing significantly in the Fe^2+^ content of the Fe_3_O_4_ particles. Two alternative exposure models were investigated; the first involving transfer of immune cell conditioned media to lung epithelial cells and the second a dual cell co-culture model comprised of 16HBE14o^−^ cells and macrophages derived from the THP-1 cell line. These alternative test systems were compared with response to dSPION induced genotoxicity in equivalent mono-culture treatments. The selection of γ-Fe_2_O_3_ and Fe_3_O_4_ dSPION as model NPs for this study was based on genotoxicity work previously undertaken on these NMs [41]. This previous study identified the genotoxic potential of dSPION to be redox dependant in a monoculture based in vitro test system, where only γ-Fe_2_O_3_ induced DNA damage. Mechanistic investigations identified that the observed DNA damage resulting from γ-Fe_2_O_3_ exposure was clastogenic as a result of oxidative stress. This is consistent with a number of studies that have highlighted SPION genotoxicity to be driven by oxidative stress in a number of in vitro and in vivo models [1, 35, 36, 40, 44].

**Table 1:** Physico-chemical characteristics of Fe_3_O_4_ and γ-Fe_2_O_3_ dSPION

	γ-Fe_2_O_3_ dSPION		Fe_3_O_4_ dSPION
Particle morphology	Cubic crystalline structure with a primary particle size of 10 nm		Cubic crystalline structure with a primary particle size of 10 nm
Chemical Composition	Fe_2_/Fe_3_ = 0.2		Fe_2_/Fe_3_ = 1.5
Hydrodynamic diameter and Zeta Potential			
Water	Size Range (nm)	37.84–531.52	11.70–164.2
	Median size population (nm)	162.2	50.75
	Polydispersal index	0.172	0.206
	Zeta Potential (mV)	−9.48 ± 0.95	−13.5 ± 1.29
10% serum medium	Size Range (nm)	18.17–220.10	28.21–141.8
	Median size population (nm)	58.77	43.82
	Polydispersal index	0.267	0.265
	Zeta Potential	−3.38 ± 0.72	−4.77 ± 1.02

γ-Fe_2_O_3_ and Fe_3_O_4_ dSPION chemical composition; hydrodynamic diameter displayed as size range (nm), median size population (nm) and polydispersity index and material zeta potential (mV)

### Mono-culture treatments

**Fig. 1:**
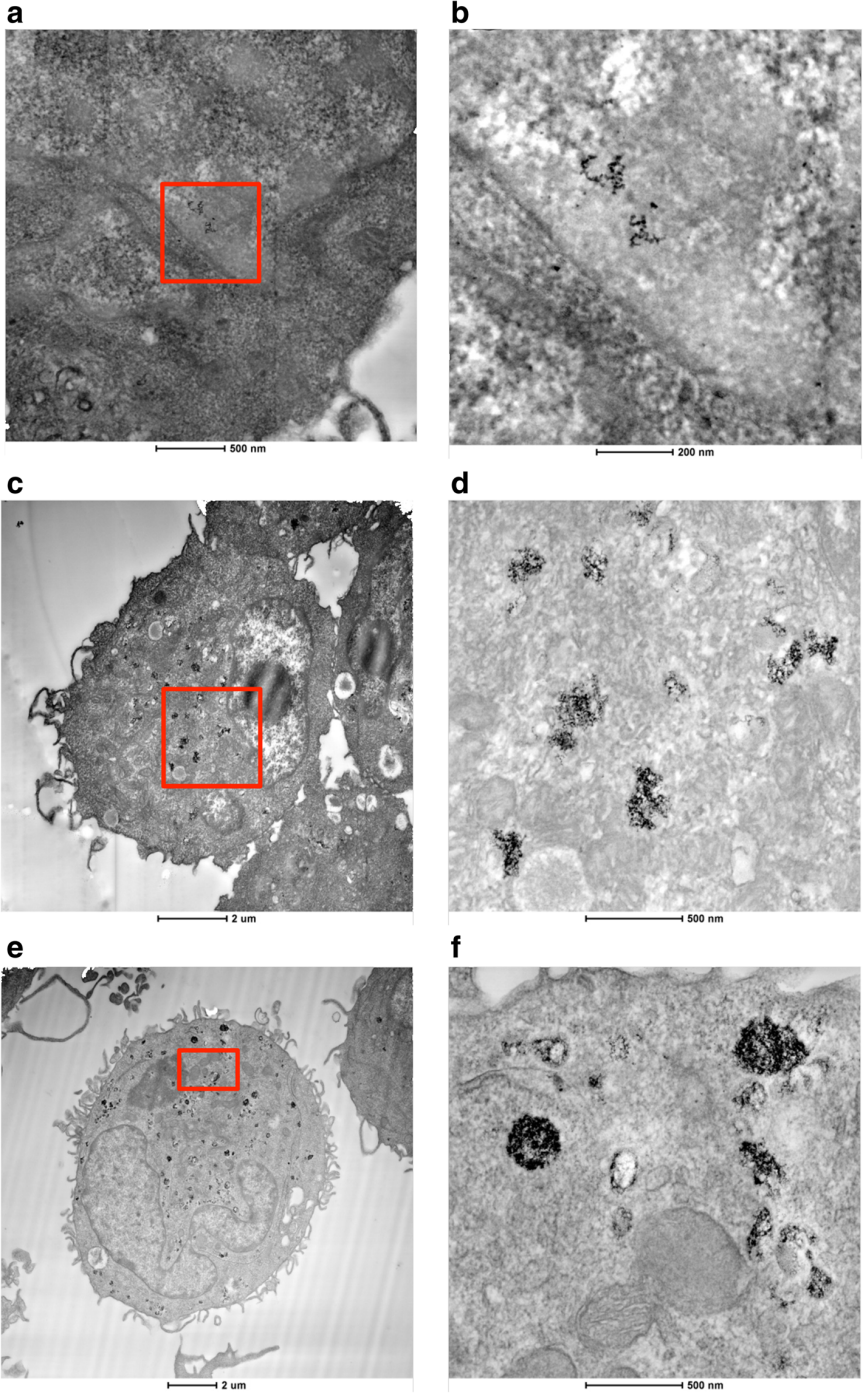
Contrast inverted HAADF STEM electron micrographs showing dSPION uptake in mono-cultured 16HBE14o^−^ cells and DTHP-1 macrophages. Uptake of γ-Fe_2_O_3_ is shown in 16HBE14o^−^ cells (**a**) and dTHP-1 macrophages (**c**). Uptake of Fe_3_O_4_ is also shown in DTHP-1 macrophages (**e**). Regions highlighted in red boxes are displayed at higher magnification in adjacent images (**b**, **d** and **f** respectively)

**Fig. 2:**
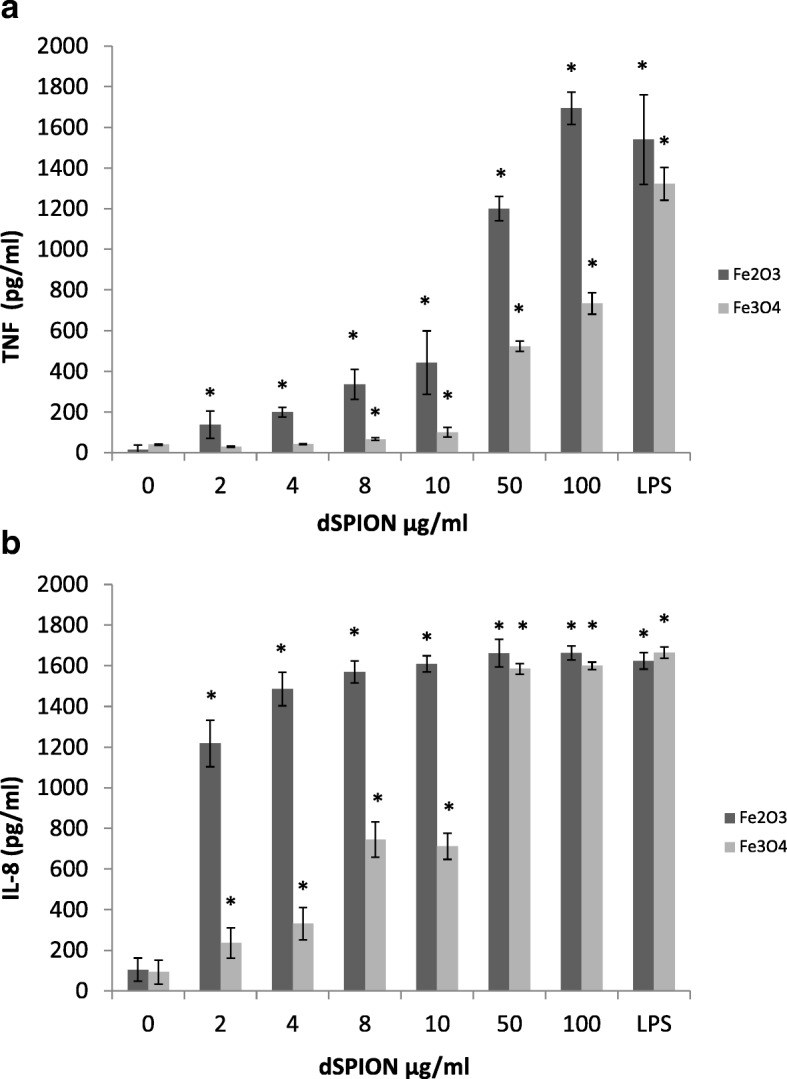
The effect of dSPION treatment on TNFα (**a**) and IL-8 (**b**) production in dTHP-1 macrophages. *p < 0.05 when compared to negative control (0 μg/ml). LPS was used as a positive control (*n* = 3)

When considering the effect of dSPION types in mono-culture treatments, neither particle type promoted statistically significant cytotoxicity in either cell type (immune cell cytotoxicity data not shown) yet nanoparticle uptake is clearly evident by TEM (Fig. 1). Both γ-Fe_2_O_3_ and Fe_3_O_4_ NPs were however able to cause an increase in (pro-)inflammatory cytokine response in both cell types. This was assessed by quantifying TNF-α and IL-8 production by dTHP-1 macrophages following treatment with γ-Fe_2_O_3_ and Fe_3_O_4_ NPs (Fig. 2 a and b respectively). There was a significant concentration-dependent increase in TNFα production (compared to the untreated control) following γ-Fe_2_O_3_ exposure at all concentrations tested following 26 h exposure (~ 1 cell cycle; Fig. 2a). A minimum 50-fold rise in TNFα levels was observed at the top three concentrations. Fe_3_O_4_ particles also showed a dose dependent increase of TNF-α from 8 to 100 μg/ml but at ~ 3 times lower levels than for γ-Fe_2_O_3_; with 50 and 100 μg/ml only resulting in a 13 and 18-fold increase over the control respectively. There was a significant increase in IL-8 production over the control at all doses of γ-Fe_2_O_3_, with a 16-fold increase observed at the 10, 50 and 100 μg/ml treatments (Fig. 2b). In comparison Fe_3_O_4_ also promoted a significant increase in IL-8 at all doses with similar 16-fold increase at the 50 and 100 μg/ml doses. There was however significantly less IL-8 quantified at the lower doses (2–10 μg/ml) compared with γ-Fe_2_O_3_ treatments. The ability of γ-Fe_2_O_3_ and Fe_3_O_4_ dSPIONs to promote a (pro-)inflammatory response in the dTHP-1 macrophages is consistent with previous studies. For example, it has been noted that PEG coated SPIONs promote a dose dependant increase in TNF production in murine macrophages [33]. Furthermore, polyethylenimine-coated SPIONs activate RAW264.7 macrophages via TLR-4 signalling, mimicking the response to LPS [29]. The ability of γ-Fe_2_O_3_ and Fe_3_O_4_ dSPIONs to promote a significant pro-inflammatory response in dTHP-1 macrophages may be directly correlated with the propensity of the NP types to be taken up by the cells, which was evident by TEM and shown to be qualitatively comparable for both cell types (Fig. 1).

**Fig. 3:**
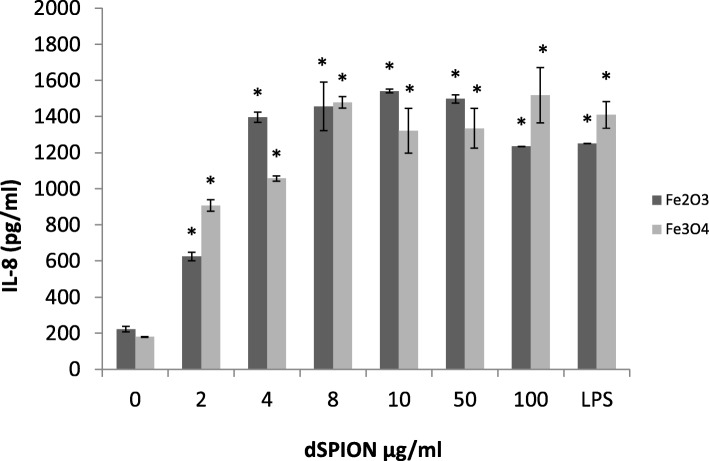
The effect of dSPION treatment on IL-8 production in 16HBE14o^−^ cells. LPS was used as positive control. *p < 0.05 when compared to negative control (*n* =. 3)

The induction of a pro-inflammatory response in 16HBE14o^−^ was assessed by quantifying IL-8 in culture supernatants following dSPION treatment (Fig. 3). IL-8 production in 16HBE14o^−^ cells following γ-Fe_2_O_3_ exposures demonstrated a significant increase in IL-8. 16HBE14o^−^ cells treated with 2 μg/ml γ-Fe_2_O_3_ over this time period showed the smallest increase over the control with only ~ 3-fold increase in IL-8 present. All other doses applied (4–100 μg/ml) demonstrated ~ 5-fold increase in IL-8 expression compared to the untreated control. Similarly, Fe_3_O_4_ dSPION treatment of 16HBE14o^−^ cells resulted in significant IL-8 expression at all doses. At 2 μg/ml, the lowest degree of IL-8 expression was induced, which represented a ~ 5-fold increase over the control. Between 2, 4 and 8 μg/ml there was a dose dependent increase in IL-8 levels, which subsequently plateaued with further concentration increases.

**Fig. 4:**
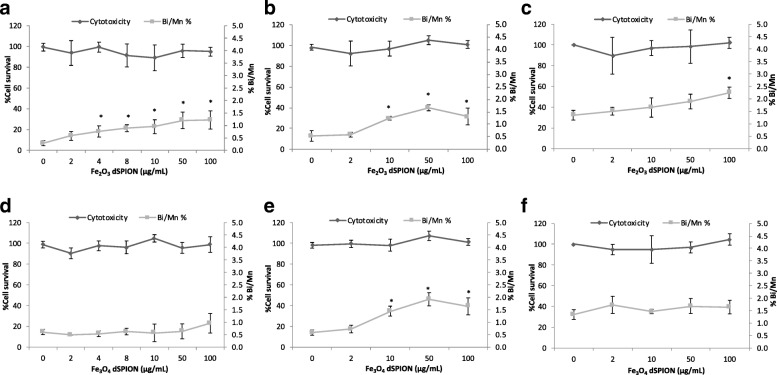
Quantification of chromosomal damage and cell viability of 16HBE14o- cells following dSPION exposure. **a** Mono-cultured 16HBE14o- cells treated with γ-Fe2O3 **b** 16HBE14o- cells treated with γ-Fe2O3 DTHP-1macrophage conditioned media **c** 16HBE14o- cells pre-treated with NAC and exposed to γ-Fe2O3 DTHP-1 macrophage conditioned media. **d** Mono-cultured 16HBE14o- cells treated with Fe3O4 **e** 16HBE14o- cells treated with Fe3O4 DTHP-1 macrophage conditioned media **f** 16HBE14o- cells pre-treated with NAC and exposed to Fe3O4 DTHP-1 macrophage conditioned media, *p < 0.05 when compared to negative control (0 μg/ml). For all CBMN assays MMC (0.01 mg/ml) was used as a positive control – (micronuclei fold increase 2.5–2.8%) (n = 3)

This similarity between the ability for both γ-Fe_2_O_3_ and Fe_3_O_4_ dSPION to cause no cytotoxicity but promote a (pro)-inflammatory response in both dTHP-1’s and 16HBE14o^−^ was not consistent in regard to the genotoxic potential in 16HBE14o^−^ cells. When undertaking CBMN assessment of γ-Fe_2_O_3_ all treatments above 2 μg/ml demonstrated statistically significant (*p* < 0.05) increases in chromosomal damage (Fig. 4a). This rose in a dose dependent manner as γ-Fe_2_O_3_ NP concentration was increased from 4 μg/ml to 50 μg/ml. The 50 μg/ml γ-Fe_2_O_3_ dose induced a 4.2-fold increase in micronucleus frequency over untreated 16HBE14o^−^ cells, which remained essentially unchanged at 100 μg/ml γ-Fe_2_O_3_ exposure too. As uptake assessment of γ-Fe_2_O_3_ in 16HBE14o^−^ cells indicated that no detectable localisation within the nucleus occurred (Fig. 1a), it can be assumed that the chromosomal breaks were promoted by indirect rather than direct means [13, 15]. Fe_3_O_4_ NPs promoted no statistically significant increase in micronucleus frequency in 16HBE14o^−^ cells observed over the full dose range applied (Fig. 4d). This redox state dependence on the ability of dSPION to cause genotoxicity in mono-cultured 16HBE14o^−^ cells is consistent with a previous study undertaken in the MCL-5 cell line [41].

### The conditioned media approach

The first approach undertaken in this study to assess the potential of NPs to promote secondary mechanism of genotoxicity was the conditioned medium approach. This process involved exposure of dTHP-1 macrophages to γ-Fe_2_O_3_ or Fe_3_O_4_ particles and the cell culture medium subsequently extracted and placed onto 16HBE14o^−^ cells. This sample preparation resulted in exposure of the 16HBE14o^−^ cells to any particle induced (pro)-inflammatory products of the macrophages, including (pro)-inflammatory cytokines and ROS/RNS. The first part of this study had indeed already demonstrated that both Fe_3_O_4_ and γ-Fe_2_O_3_ dSPIONs induce a significant (pro)-inflammatory response in dTHP-1 cells (Fig. 2).

Initially, chromosomal damage was assessed by the CBMN assay in the 16HBE14o^−^ cell line following 22 h treatment with immune cell conditioned media derived from pre-exposure of THP-1 macrophages to γ-Fe_2_O_3_ and Fe_3_O_4_ (Fig. 4b and e respectively). Cytotoxicity evaluation (RPD) was also performed alongside which was observed to be insignificant for both NP types. Both γ-Fe_2_O_3_ and Fe_3_O_4_ dSPIONs promoted a similar induction of chromosomal breakage at the 10, 50 and 100 μg/ml treatments. This is in contrast to the fact that Fe_3_O_4_ has been identified as non-genotoxic when applying the NPs directly to 16HBE14o^−^ cells (Fig. 4d).

To assess the potential role of oxidative stress in the genotoxicity response observed, cells were pre-treated with NAC a precursor in the formation of the antioxidant, glutathione (GSH) [27]. Upon doing so a reduction in micronuclei frequency was observed for both γ-Fe_2_O_3_ and Fe_3_O_4_ exposures (Fig. 4c and f respectively). A higher background micronuclei frequency following NAC pre-treatment was noted, however this was deemed to be within acceptable limits [32]. As NAC is an anti-oxidant precursor this result indicated that oxidative stress was potentially a factor involved in driving the DNA damage observed in the cell conditioned media exposures. The most prominent mechanism by which a NM may induce macrophage ROS production is via macrophage NADPH activation resulting in an oxidative burst [4]. It is highly unlikely however that macrophage produced O_2_^−^ and OH• would have been transferred to the 16HBE14o^−^ cells in this study as these ROS have a limited half-life of 10^− 9^ s [12]. However other more stable oxidative mediators maybe transferred, which in combination with Fe^2+^ ions could permit the formation of OH• during subsequent 16HBE14o^−^ cellular exposure [43]. It is also probable that dTHP-1 (pro)-inflammatory cytokine transfer would cause the activation of a (pro)-inflammatory response in the 16HBE14o^−^ cell. This effect in itself may have been a promotor of DNA damage in the 16HBE14o^−^ cells by the promotion of nitric oxide synthase assembly [23].

The use of immune cell conditioned media offers the advantage of being a relatively simplistic approach that could easily be applied to standard in vitro techniques for genotoxicity evaluation, allowing techniques such as the CBMN assay to be used without the need for further adaptation. The approach allows the transfer of (pro)-inflammatory mediators to other cell types, simulating the response of immune cell interaction in vivo. The use of conditioned media however could be regarded as a crude approach due the fact that it does not permit cellular interplay and that during the transfer process a significant number of potential genotoxic mediators would be lost [16].

### Co-culture models

The next level of increased in vitro model complexity is the combination of different cell types to form a co-culture system. This approach allows for cellular interplay and exposure to short lived potential genotoxic mediators, which are unachievable using the conditioned media technique. For this investigation, a dual cell co-culture model comprised of 16HBE14o^−^ cells and dTHP-1 macrophages was constructed as a representation of lung tissue. The co-culture was inspired by the model developed by Rothen-Rutishauser et al. [37], which comprised of a lung epithelial cell layer with human blood monocyte derived macrophages on the apical side, and dendritic cells on the basal side.

**Fig. 5:**
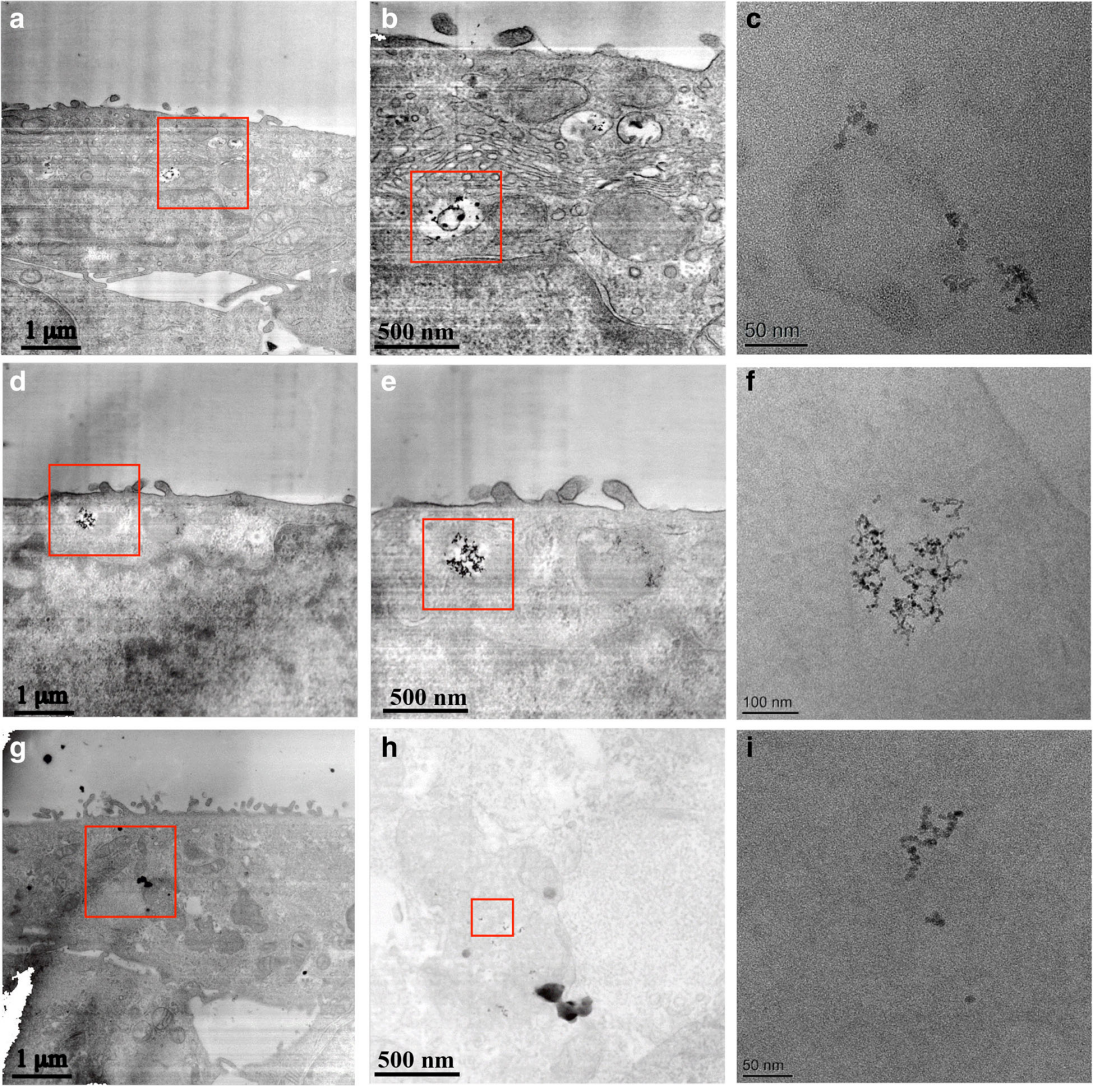
TEM/STEM micrographs of 16HBE14o-/DTHP-1 lung co-culture model following treatment with dSPION – (**a**) – inverted contrast HAADF STEM image of γ-Fe2O3 internalised within DTHP-1 macrophage on top of a 16HBE14o- epithelium cellular layer – region in red box in shown in (**b**) and higher magnification bright field TEM shown in (**c**); note the ~ 90o clockwise rotation of the TEM image. (**d**) - STEM image of γ-Fe2O3 internalised by 16HBE14o-, cell region highlighted by red box is shown in (**e**) and higher magnification TEM is shown in (**f**); TEM image is rotated clockwise relative to STEM image. (**g**) - STEM image of Fe3O4 within DTHP-1 macrophage within the co-culture – region in red box in shown in (**h**) and high magnification TEM shown in (**i**)

The currently presented lung co-culture model was initially applied to assess dSPION uptake by electron microscopy (Fig. 5). This was a critical step to evaluate if and where SPION localisation occurred within the model [8]. Various studies have shown different NMs to penetrate into human lung cells both in vivo and in vitro [6, 17, 30]. Uptake of both dSPION types by dTHP-macrophages was observed, with both NPs seen either within membrane bound vesicles or free within the cytoplasm. In the 16HBE14o^−^ cells γ-Fe_2_O_3_ was located in membrane bound vesicles, suggesting that the uptake into this cell type was via endocytosis. Due to the lack of any detectable nuclear uptake (as with mono-cultured 16HBE14o^−^ cells, Fig. 1a) it is suggested that no direct interaction occurred between the cells genetic machinery and the NPs. In contrast, no Fe_3_O_4_ NPs were identified inside the 16HBE14o^−^ cellular component of the co-culture model. However, it should be noted that this investigation of uptake by electron microscopy was qualitative and not quantitative.

**Fig. 6:**
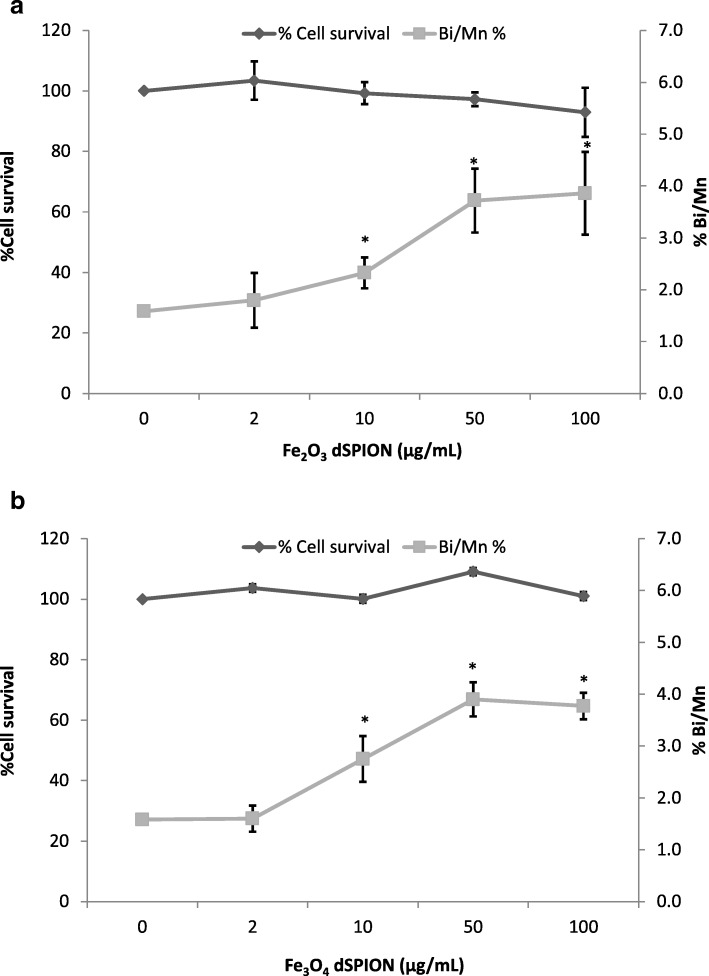
Chromosomal damage and toxicity assessment of dSPION treated 16HBE14o^−^ cells within the lung co-culture model **a** Co-culture model treated with γ-Fe_2_O_3_ dSPION and **b** co-culture model treated with Fe_3_O_4_ dSPION both for 22 h. *p < 0.05 when compared to negative control (0 μg/ml). *MMC (0.1 μg/ml) used as positive control (micronuclei frequency 4.01%) (n = 3)*

The DNA damaging potential of γ-Fe_2_O_3_ and Fe_3_O_4_ dSPIONs when applied to the 16HBE14o- and THP-1 lung co-culture models for 22 h was assessed by the CBMN assay with CBPI analysis undertaken alongside to evaluate cytotoxicity. Exposure of the co-culture model to γ-Fe_2_O_3_ resulted in no significant promotion of cytotoxicity significance (Fig. 6a). Chromosomal damage assessment in 16HBE14o- cells did however result in a significant increase in micronucleus frequency at the 10, 50 and 100 μg/ml treatments. Fe_3_O_4_ dSPIONs also did not induce cytotoxicity in the 16HBE14o- cells and also demonstrated a similar dose response profile when chromosomal damage was assessed (Fig. 6b).

Due to the similarity between the chromosomal damage observed in the mono-cultures, co-cultures and conditioned media treatments with γ-Fe_2_O_3_ dSPION, it is likely that the DNA damage observed was due to a combination of both primary and secondary mechanisms. Fe_3_O_4_ dSPION however, differed significantly in that no chromosomal damage was induced in mono-cultured 16HBE14o^−^ cells, but DNA damage was observed in both conditioned media and co-culture treatments (*p* < 0.05). This means that DNA damage induction by Fe_3_O_4_ dSPION is only promoted by secondary mechanisms i.e. due to nanoparticle interactions with dTHP-1 macrophages. Secondary DNA damaging mechanisms may not be limited to the induction of a macrophage oxidative burst, as macrophage production of (pro)-inflammatory cytokines may also play a role. Cytokines such as TNFα and IL-β can cause nitric oxide synthase assembly within epithelial cells resulting in the production of intercellular nitric oxide [19]. Indeed, this is supported by the mono-culture components of this study demonstrating that the test SPIONs both promote activation of TNF-α and IL-8 in dTHP-1 macrophages (Fig. 2).

To the best of the authors’ knowledge there are no reports of other in vitro co-culture models that have demonstrated SPIONs or any NM to promote DNA damage by secondary mechanisms, therefore direct comparison is difficult. Studies have been undertaken to assess the genotoxicity of particulate matter in vitro using co-culture models such as that by Jantzen and colleagues [21]. However, this study noted a decrease of genotoxicity in co-cultures compared to mono-culture treatments, therefore secondary genotoxicity was not evident following diesel exhaust exposure. This current in vitro SPION study offered the significant advantage over previous investigations as Fe_3_O_4_ dSPION clearly promoted a genotoxic response in exposure scenarios that incorporated macrophages (co-culture), but not in the monoculture based system. There are however various in vivo NM genotoxicity studies that attribute observed DNA damage to chronic pulmonary inflammation. For example, carbon black instillation in mice lung resulted in a chronic inflammatory response and subsequent secondary oxidative DNA damage [7]. Moreover, chronic exposure to silica has directly been attributed to irreversible pulmonary inflammatory disease resulting in lung tumour growth in mice [39]. A recent study aiming to provide insight into the mode of action of DNA damage caused by 15 nm silica particles in rat livers highlighted that observed DNA damage was a direct consequence of oxidative damage caused by the initiation of an immune response in the tissue [34]. These in vivo studies are supportive of the principle demonstrated in this investigation; that secondary genotoxicity promoted by NM-immune cell interaction should be an important consideration when assessing the DNA damage potential of NMs. Current standard in vitro DNA damage tests only evaluate primary genotoxicity due to reliance on single cell based systems. While these studies have provided vital data supporting hazard evaluation, they may not represent the complete array of damage mechanisms that could occur in vivo. Consequently, monoculture based systems for NM genotoxicity assessment need to be replaced with approaches that allow the incorporation of secondary genotoxic mechanisms that maybe inferred to operate in vivo. At its most simplistic this can be achieved by the conditioned media approach shown here. However, to more fully mimic mechanisms promoted within tissues more complex systems such as co-culture models need to be widely developed and utilised to provide more representative and informative assessments of the risks posed by NM exposures.

## Conclusions

This study has demonstrated the limitations of solely utilising mono-cell culture based test systems for evaluating SPIONs and NM genotoxicity in general, in vitro*.* We show here that both γ-Fe_2_O_3_ and Fe_3_O_4_ SPIONs can induce secondary genotoxicity in 16HBE14o- epithelial cells via interaction with immune cells yet only the γ-Fe_2_O_3_ ellicit this response in 16HBE14o- monocultures. Moving forward, the field needs to further develop alternative in vitro strategies to incorporate the concept of multi-cell interactions to better replicate the potential of DNA damage induction by secondary mechanisms.

## 3. Materials and methods

### Materials

dSPIONs in both γ-Fe_2_O_3_ and Fe_3_O_4_ states, dispersed in water at 10 mg/ml were purchased from Liquids Research, Bangor, UK. Both dSPION types were shown to be negative for endotoxin contamination using the E-TOXATE gel clot assay (Sigma, UK) (data not shown). All other chemicals or reagents were purchased from Sigma-Aldrich (Gillingham, UK) unless otherwise stated.

### Cell culture

The bronchial cell line, 16HBE14o- (kindly donated by Dr. Grunet, University of California, San Francisco) was cultured in Minimum Essential Medium (MEM) (with 10% L-glutamine) supplemented with 10% Fetal Bovine Serum (FBS) and 1% streptomycin/penicillin. All culture surfaces for this cell line were coated with fibronectin solution; 88% LHC basal medium, 10% 1 mg/ml Bovine Serum Albumin (BSA), 1% 3 mg/ml bovine collagen and 1% 1 mg/ml human fibronectin. THP-1 monocyte cells were purchased from American Type Culture Collection (Manassas, VA, USA) and cultured in RPMI 1640 supplemented with 10% FBS, 1% L-glutamine and 1% streptomycin/penicillin. Both cell lines were incubated at 37 °C with 5% CO_2_. Differentiation to macrophage-like cells (dTHP-1) was achieved by supplementing 5 × 10^5^ cells/ml in 10 ml media with 20 nM phorbol 12-myristate 13-acetate (PMA) dissolved in dimethyl sulfoxide (DMSO) for 24 h, adhered cells were given 24 h recovery in fresh complete media prior to treatment. Prior to cell culture treatments γ-Fe_2_O_3_ and Fe_3_O_4_ dSPION stocks (10 mg/ml) were briefly vortexed then diluted in cell culture media to the required exposure levels then added directly to cell cultures via pipetting. All experiments were performed in triplicate (*n* = 3).

### Physico-chemical characterisation

All physico-chemical characterisation of γ-Fe_2_O_3_ and Fe_3_O_4_ dSPION was undertaken at a concentration of 100 μg/ml as a representation of the highest dose used during cell culture treatments. Full physico-chemical characterisation of both γ-Fe_2_O_3_ and Fe_3_O_4_ dSPIONs was undertaken to assess particle size, shape, chemical composition, surface charge and agglomeration state when dispersed in water and cell culture media (Table 1).

Transmission electron microscopy (TEM) was used to analyse particle size, shape, morphology, crystallinity and purity of both γ- Fe_2_O_3_ and Fe_3_O_4_ dSPIONs. A drop of diluted material was drop-cast on a copper TEM grid coated with a continuous carbon film and left to air dry. TEM analysis was undertaken with a FEI Tecnai F20 TEM operating at 200 kV and fitted with a Fischione high angle annular dark field (HAADF) detector, a Gatan Orius SC600A CCD camera, and an Oxford Instruments 80 mm^2^ silicon drift energy-dispersive X-ray (EDX) spectrometer.

Agglomerate medial and size distribution of dSPION samples was determined by dynamic light scattering (DLS) using a Malvern Zeta-Sizer (Malvern instruments Ltd., UK). Measurements were performed in water and MEM with 10% FBS and presented as an average of 10 readings, with samples briefly vortexed and incubated at 37 °C prior to measurements. Particle zeta potential was determined by injecting 500 μl of appropriately dispersed particle suspension into a Folded Capillary Cell (Malvern, UK) using a 1 ml syringe. The capillary cell was placed into a ZetaSizer (Malvern, UK) and allowed to equilibrate for 2 min before measurement was initiated. Each reported measurement is an average of 10 scans and each sample was run in duplicate from separate preparations. The dispersant dielectric constant was set at 74.5 Hz and Henry’s function set at the Smoluchowski approximation of F(κα) = 1.5. The oxidation state of both dSPIONs was confirmed by X-ray Photoelectron Spectroscopy (XPS) as previously detailed in [41].

### TEM to assess cellular uptake of dSPION

γ-Fe_2_O_3_ and Fe_3_O_4_ dSPION cellular uptake in both mono-culture cells and in co-cultured models (grown on membrane inserts) was confirmed by TEM imaging (FEI Tecnai F20). Mono-cultured dTHP-1 cells and 16HBE14o^−^ cells were treated with 100 μg/ml γ-Fe_2_O_3_ and Fe_3_O_4_ for 26 h and 22 h respectively. Co-culture models were treated with equal doses for 22 h. Fixing, embedding, sectioning and imaging was undertaken as previously described in [48].

### Enzyme-linked immunosorbent assay (ELISA) for TNF-α and IL-8

Supernatants from 16HBE14o^−^ cells and dTHP-1 macrophages following treatment to both γ-Fe_2_O_3_ and Fe_3_O_4_ dSPIONs were analysed by TNF-α and IL-8 ELISA’s (DuoSet ELISA; R&D Systems Europe). All experiments were performed in triplicate (*n* = 3) following the manufacturer’s instructions and NP exposure was undertaken for 26 and 22 h for dTHP-1 and 16HBE14o^−^ cells respectively (to allow consistency between uptake, cytotoxicity and genotoxicity assessment). Lipopolysaccharide (LPS) was used as a positive control.

### dTHP-1 macrophage cytotoxicity

Cytotoxicity of dTHP-1 macrophages was assessed by the trypan blue exclusion assay [45]. Cells were treated with γ-Fe_2_O_3_ and Fe_3_O_4_ dSPION for one cell cycle (26 h). Following treatment cells were detached using Accutase, exposed to trypan blue (1:5 dilution) and all live cells scored using a haemocytometer (*n* = 3).

### In vitro cytokinesis blocked micronucleus (CBMN) assay of mono-cultured 16HBE14o^−^

16HBE14o^−^ cells were seeded at 1 × 10^5^ cells/ml and allowed to adhere for 22 h after which the cells were then treated with dSPIONs for 22 h (ca. 1-cell cycle). Mitomycin-C (MMC) at 0.01 μg/ml was used as a positive control. After exposure, cells were washed in PBS 3 times and re-suspended in fresh media containing 3 μg/ml cytochalasin B for a further 22 h. The cells were then trypsinised, pelleted by centrifugation and washed twice in PBS. Slides were prepared and scored for the presence of micronuclei in binucleated cells using the automated micronucleus Metafer image analysis system (Metasystems, Carl Zeiss Ltd) as described previously in [41]. All experiments were performed in triplicate (*n* = 3) and 1000 cells per replicate were scored (3000 in total for each treatment).

### Conditioned medium treatments

dTHP-1 macrophages were treated with both dSPIONs for 26 h (1-cell cycle), the dTHP-1 medium was then removed and ultra-centrifuged to discard the excess nanoparticles. 16HBE14o^−^ cells were treated with the nanoparticle-free conditioned media for 22 h (1-cell cycle) and the CBMN assay was performed. To assess the role of oxidative stress in promoting DNA damage, 16HBE14o^−^ cells were pre-treated with 2 mM of the antioxidant N acetyl-L-Cysteine (NAC) for 2 h prior to treatment with conditioned media [41]. The CBMN assay was then performed as described above.

### Construction of co-culture lung model

Co-culture lung models were comprised of 16HBE14o^−^ cells and dTHP-1 macrophages. The models were constructed on 4.2 cm^2^ trans-well inserts with a PET membrane (3 μm pores) that were supported in a 6-well companion plate (Corning, Germany). Prior, to cell culture the apical side of the trans-well membranes were pre-treated with fibronectin solution. The first stage of the co-culture construction required the establishment of a stable 16HBE14o^−^ epithelium on the apical side of the fibronectin coated trans-well by seeding at a concentration of 1 × 10^6^ cells/ml for 7 days at 37 °C with 5% CO_2_. Subsequently, 500 μl of dTHP-1 cells (1 × 10^5^ cells/ml) were placed on to the 16HBE14o^−^ epithelial layer and allowed to adhere for 1.5 h. Following this step any excess macrophages in suspension were removed and replaced with 2 ml of fresh MEM culture medium. Co-culture models were allowed to further establish for 24 h prior to use (co-culture model structure is illustrated in Additional file 1: Figure S1) [37].

### Co-culture in vitro CBMN assay

Co-culture treatments were undertaken for 22 h (ca. 1x 16HBE14o- cell cycle). Cultures were then washed in PBS and media containing 3 μg/ml cyto-B was placed in both the upper and lower trans-well chambers and incubated for 22 h. Cells were subsequently trypsinised, fixed in 3% paraformaldehyde and permeabilised with Triton X100. Cells were washed with PBS prior to staining with 1 μg/ml of anti-human CD324 (e-cadherin) with a conjugated FITC fluorophore (BioLegend®, San Fransico). Following washing and resuspension in 1 ml of PBS, cells were pipetted on to slides and coverslips were attached with DAPI VECTASHIELD (VECTOR Laboratories, USA). Cell imaging and micronuclei identification was undertaken using an Axioimager Z2 fluorescent microscope with a one megapixel charged coupled device camera (Carl Zeiss, UK). For micronucleus identification 500 binucleated cells per replicate were scored (in total 1500 binucleated cells per dose); binucleated cells were confirmed as 16HBE14o- by the presence of CD324-FITC fluorescence upon exposure to light of wavelength 488 nm. Only cells that showed a fluorescent signal were considered epithelial cells and therefore counted. Cells that did not demonstrate CD324-FITC binding were presumed to be macrophages and disregarded. Cytotoxicity was assessed alongside micronucleus scoring by the cytokinesis-blocked proliferation index (CBPI) as described previously [25].

### Statistical analysis

All data are presented as the mean +/− standard deviation. All statistical testing was performed by one Way ANOVA with Dunnet’s post hoc testing (SPSS v22.0, Chicago). Differences were deemed significant when *p* < 0.05. Statistical analysis comparing micronucleus fold change was undertaken between mono-culture/conditioned media treatments and mono-culture/co-culture treatments for each dSPION type by one way ANOVAs.

## Additional file

Additional file 1: Structural characterisation of dTHP-1/16HBE14o co-culture model (A) Laser scanning microscopy images of co-culture mode demonstrating the dTHP-1 macrophage layer (stained with C11b antibody with a FITC conjugate and DAPI) on top of the 16HBE14o- epithelium (stained with a CD324 antibody with an Alexa Flour® 647 conjugate). (B) TEM image of dTHP-1 macrophage on top of 16HBE14o- epithelium. (PDF 349 kb)
