# Supplementary material for: In vitro detection of in vitro secondary mechanisms of genotoxicity induced by engineered nanomaterials
Source: Part Fibre Toxicol. 2019 Feb 13;16:8. doi: 10.1186/s12989-019-0291-7 (PMC6374901; doi:10.1186/s12989-019-0291-7)
Supplement: Supplementary file 1 — Structural characterisation of dTHP-1/16HBE14o co-culture model (A) Laser scanning microscopy images of co-culture mode demonstrating the dTHP-1 macrophage layer (stained with C11b antibody with a FITC conjugate and DAPI) on top of the 16HBE14o- epithelium (stained with a CD324 antibody with an Alexa Flour® 647 conjugate). (B) TEM image of dTHP-1 macrophage on top of 16HBE14o- epithelium. (PDF 349 kb) [file 12989_2019_291_MOESM1_ESM.pdf]

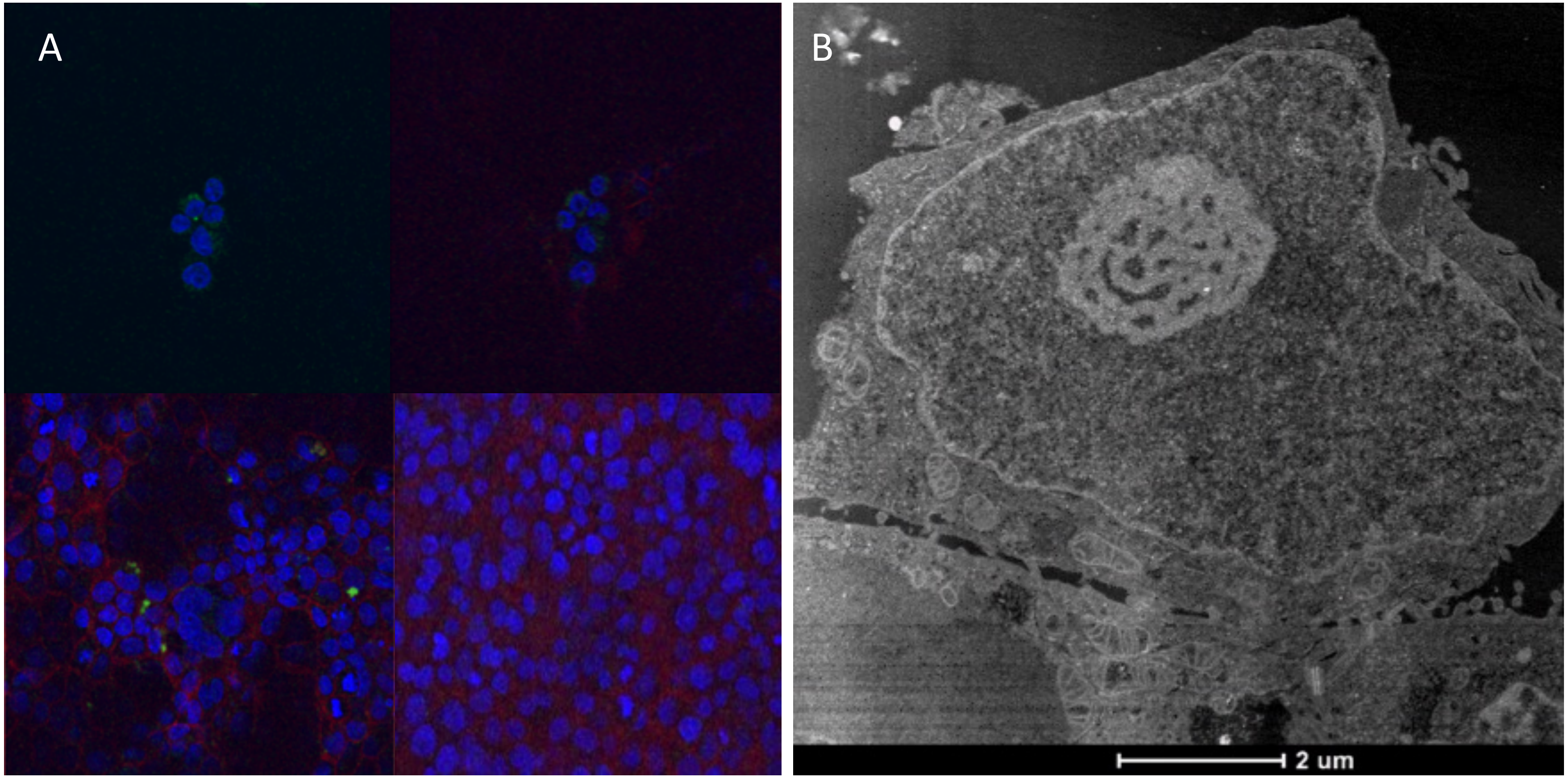

S1 – Structural characterisation of dTHP-1/16HBE14o co-culture model (A) Laser scanning microscopy images of co-culture mode demonstrating the dTHP-1 macrophage layer (stained with C11b antibody with a FITC conjugate and DAPI) on top of the 16HBE14o^{-} epithelium (stained with a CD324 antibody with an Alexa Flour® 647 conjugate). (B) TEM image of dTHP-1 macrophage on top of 16HBE14o^{-} epithelium.
